# Comment on “The Critical Omission of CRRT Dose in Comparative Studies of RRT Modalities for Sepsis-Associated AKI”

**DOI:** 10.1186/s40981-025-00804-w

**Published:** 2025-08-09

**Authors:** Hiromu Okano, Hiroshi Okamoto, Haruna Tanaka, Ryota Sakurai, Tsutomu Yamazaki

**Affiliations:** 1 Department of Critical Care Medicine, St. Luke’s International Hospital, 9-1 Akashi-Cho, Chuo-Ku, Tokyo, 104-8560 Japan; 2 Department of Social Medical Sciences, Graduate School of Medicine, International University of Health and Welfare, Minato City, 4-1-26 Akasaka, Tokyo, 107-8402 Japan; 3 Department of Biostatistics, Division of Health Sciences and Nursing, Graduate School of Medicine, The University of Tokyo, 7-3-1 Bunkyo-Ku, Tokyo, 113-8655 Japan

To the Editor,

We appreciate the insightful comments by Wang and Yao [1] regarding our recently published study comparing intermittent renal replacement therapy (IRRT) and continuous renal replacement therapy (CRRT) for sepsis-associated acute kidney injury (S-AKI) [2]. We agree that the absence of detailed CRRT dosing parameters—such as effluent rate and downtime—is a limitation of our study, and we recognize that CRRT dose is a critical factor in interpreting clinical outcomes.

As Wang et al. [1] correctly noted, the Kidney Disease: Improving Global Outcomes (KDIGO) guidelines recommend a CRRT dose of 20–25 mL/kg/h [3]. However, actual practice in Japan often falls below this threshold due to constraints imposed by the national healthcare reimbursement system, in which the use of replacement and dialysis fluids is typically limited to 10–16 mL/kg/h (15–24 L/day), and reimbursement is provided as a fixed daily fee regardless of effluent volume. This structure has discouraged higher-dose prescriptions and led to the widespread adoption of lower-dose CRRT in clinical practice [4–6]. Notably, the study by Yasuda et al. found that even lower-intensity CRRT (~ 10–15 mL/kg/h) could achieve adequate uremic control in Japanese ICU patients with severe AKI [6].This finding provides context for our cohort, in which the actual CRRT intensity may have been lower than international guidelines, yet likely sufficient for basic solute clearance.

We also emphasize that in resource-constrained settings, the primary goal of CRRT—particularly in septic shock—is often hemodynamic stabilization, rather than maximal solute removal. From this standpoint, lower-dose CRRT may be seen not as inherently suboptimal, but rather as a practical and targeted approach aligned with clinical realities.

Nonetheless, we concur that future research should more explicitly report CRRT dose parameters and assess dose–response relationships in S-AKI populations. Our study, conducted under real-world conditions in Japanese ICUs, provides complementary evidence that both IRRT and CRRT, as practiced, may be similarly effective in terms of in-hospital mortality.

In conclusion, while we acknowledge the limitation of not reporting CRRT dose details, we believe our study offers clinically relevant insights reflective of current Japanese ICU practices. We hope this encourages further research into optimal CRRT dosing strategies within diverse healthcare systems.

## Data Availability

The datasets generated in this study are available from the corresponding author upon request.

## Abbreviations

CRRT: Continuous renal replacement therapy
IRRT: Intermittent renal replacement therapy
S-AKI: Sepsis-associated AKI
